# Supplementary material for: An ultrafast plenoptic-camera system for high-resolution 3D particle tracking in unsegmented scintillators
Source: Nat Commun. 2026 Mar 21;17:4204. doi: 10.1038/s41467-026-70918-x (PMC13156296; doi:10.1038/s41467-026-70918-x)
Supplement: Supplementary file 1 — Supplementary Information [file 41467_2026_70918_MOESM1_ESM.pdf]

# – Supplementary Information –
# An ultrafast plenoptic-camera system for high-resolution 3D particle tracking in unsegmented scintillators

Till Dieminger[1], Saúl Alonso-Monsalve[1], Christoph Alt[1], Claudio Bruschini[2], Noemi Bührer[1], Edoardo Charbon[2], Kodai Kaneyasu[2], Tim Weber[1], Matthew Franks[1], Davide Sgalaberna[1*]

[1]IPA, ETH Zürich, Otto Stern Weg 5, Zurich, 8093, Zurich, Switzerland.
[2]Advanced Quantum Architecture Lab (AQUA), EPFL, Rue de la Maladière, 2000, Neuchâtel, Switzerland.

*Corresponding author(s). E-mail(s): davide.sgalaberna@cern.ch;

## Supplementary Methods

### 1. Optical model and simulation

The interaction and propagation of particles in the scintillator volume were simulated with the Geant4 toolkit [1–3]. The parameters of polyvinyltoluene-based plastic scintillator are simulated with light yield (8,700 photons / MeV), emission and absorption spectra, attenuation length (2.5 m), refractive index (1.58), and decay time ($\sim$ 2.1 ns) characteristic of EJ-262 [4], the scintillator used for the PLATON-prototype. It is worth mentioning that the purpose of this simulation study is general and is applicable to a wide variety of organic scintillators, including those in the form of liquid, often used in giant neutrino experiments [5, 6]. Their typical light yield is around 10,000 photons / MeV, attenuation lengths equal or above 20 m, as well as very high purity, thus, a very uniform optical performance.

Neutrinos are simulated as follows. The neutrino energy was simulated according to the flux made public by the T2K experiment, where it is used in the neutrino oscillation data analyses [7, 8]. The flux is peaked at 0.6 GeV and its broad band ranges mainly from 0.2 to 1.5 GeV, with a long tail up to 30 GeV. The T2K neutrino angular distribution is wide, with a standard deviation of about 4 meters at the near detector location [9]. Thus, for simplicity we decided to simulate neutrinos on a fixed direction, parallel to both sensor planes, without loss of generality. The NEUT 5.5.0 neutrino event generator [10], used at the T2K experiment for neutrino data analyses, is used to simulate the neutrino interactions in the scintillator. It provides the list of final-state particles (particle types and corresponding momentum vectors) and the 3D position of the neutrino interaction vertex. Then, the propagation of the final-state particles in matter is simulated using the Geant4 toolkit [1–3]. We assume the distance between the position of the ionising particle and the final emission of the scintillation light to be small compared to the 3D spatial resolution of the PLATON camera [11, 12] and, thus, no additional smearing on the position of the scintillation light emission is applied. The optical photons produced by the scintillation process are propagated using native Geant4 optical photon processes, including absorption and reflections

following the Fresnel equations. The speed of photons in scintillator is about 20 cm / ns. Once the photons leave the scintillator volume, their positions and directions are stored for subsequent processing steps.

The second part of the optical simulation is based on a custom library that simulates lenses, sensors, and refractive surfaces outside the scintillator volume. It performs the ray tracing of each individual photon from the exit point on the scintillator surface to the photosensor, through the plenoptic system. Thin and thick lenses, together with their apertures, are implemented in the paraxial approximation, assuming 100% lens transparency and neglecting optical aberrations. The propagation time of each photon is computed assuming straight-line travel between optical elements and using the speed of light in vacuum. Further, general surfaces can be added to the optical stack by defining their shape using tessellated triangles in an STL file.

The components of the plenoptic camera model include: the MLA, parametrised as an array of thin lenses or as an array of pinholes with constant same pitch; the parameters of the MLA thin-lens model, including the diameter ($D_\mu$) and the lateral position ($L_\mu$) of each micro-lens; and the distance between the MLA and the sensor planes ($B$) and the focal length of the MLA ($f_{MLA}$). The geometry of the MLA accounts for the insensitive areas between adjacent micro-lenses. All the micro-lenses have the same diameter and focal length. The main lens is modelled with the thick-lens equation, which includes its diameter ($D_L$), the position of the two principal planes ($P_1$ and $P_2$), and the focal length $f_L$. This model can be easily converted to that of a thin lens if the two parallel principal planes coincide.

The simulation of the SPAD array sensors was based on the parameters of SwissSPAD2 [13]. The pixel active and dead areas of the SPAD array were simulated as designed, thus with a geometrical fill factor. The photon detection efficiency (PDP) was simulated as a binomial efficiency following the corresponding wavelength spectrum. The DCR was also cross-checked against measurements performed in our laboratory, and where explicitly mentioned, extrapolated to a future, realistic performance. The timestamp of each pixel can be simulated in either self-trigger or gate mode.

The positions and the relative distances of the optical objects were adjusted to reflect the configuration of the PLATON-prototype (see the “Image post-processing method” and “Calibration of the PLATON-prototype” subsections of Methods of the main text) or that of an optimised PLATON detector (see the “Simulated particle detection with the PLATON-10cm detector” subsection of Results of the main text). Although this model appears rather simple, it accurately reproduces the actual resolution of the PLATON-prototype.

## 2. Data-driven likelihood reconstruction analysis

Alternative post-processing methods were also tested. The first one consists of a data-driven likelihood analysis. The intensity greyscale images for each of the 160 points scanned during the calibration campaign were used as probability density functions (PDFs). To each PDF, the distribution of the dark counts across the pixels, obtained with an independent measurement, is subtracted. This way we could estimate the intrinsic spatial resolution of the plenoptic system for the case of a negligible contamination from dark counts. This study aims to emulate the scenario of a future SPAD array chip capable of rejecting dark counts by time coincidence by constructing sampled data frames: after subtracting the independently measured dark-count map, we generate low-photon-count binary frames by sampling from the resulting intensity PDFs. See more details in Discussion of the main text. “Sampled” data frames were obtained by randomly throwing the position of the counts from the PDF of a given position of the light source.

In a given sampled frame, each pixel $i$ can take the value $n_i = 0$ or 1, following the Bernoulli probability distribution. On the other hand, the probability $p_i(\vec{y})$ for a pixel to take the value of 1 depends also on the position of the point light source $\vec{y}$ and is described by the corresponding measured PDF. The corresponding negative log-likelihood is given by the binary cross-entropy ($BCE$), which compares a sampled data frame taken at the position $\vec{x}$ to the PDF at a position

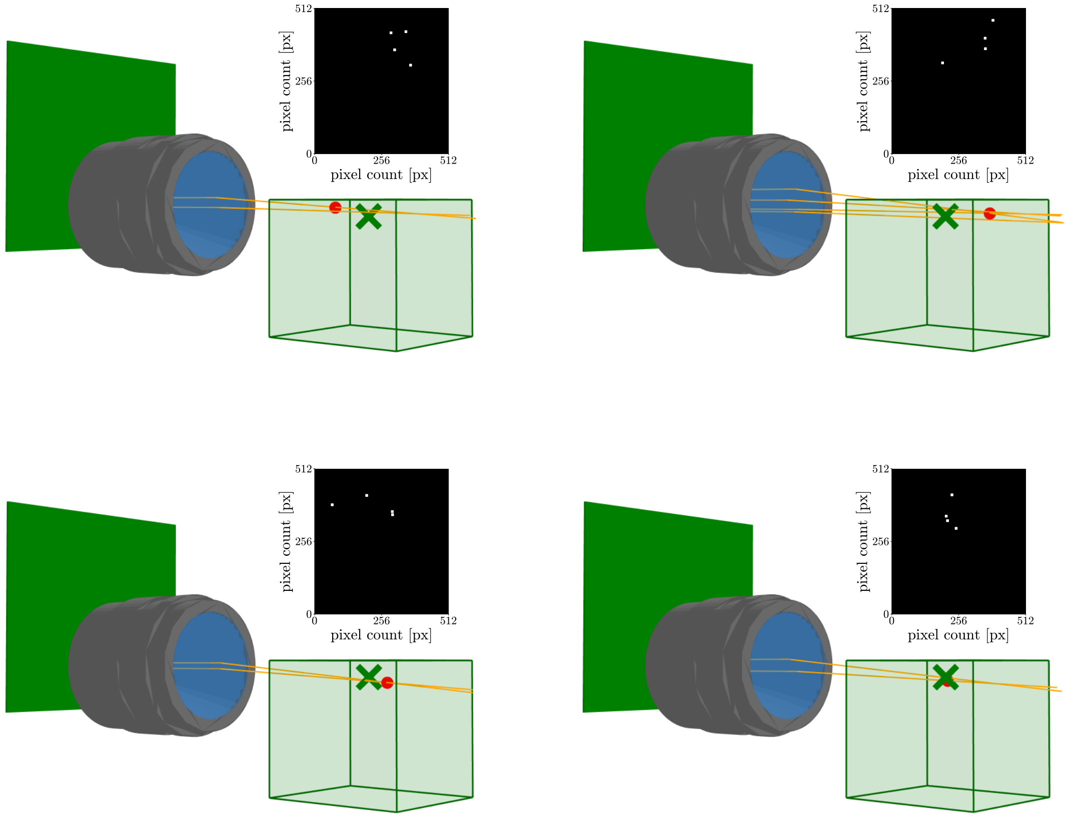


**Supplementary Fig. 1**: **Four event displays of reconstructed $^{90}$Sr electron candidates.** Shown are the four identified events, with 4 non masked initial counts on the sensor. For each event the image on the sensor is show, with enlarged active pixels for visibility. Below each event is shown in an event display, where in orange, the reconstructed rays are visible, together with the real source location (green cross) and the reconstructed point of the $^{90}$Sr (red circle). The data analysis is described in the "3D spatial resolution for a point-like light source" subsection of Results of the main text.

$\vec{y}$ and is given by

$$BCE(I_{\vec{x}}|\vec{y}) = -\frac{1}{N_{\text{pixels}}}\sum_{i}^{N_{\text{pixels}}} n_i \log(p_i(\vec{y})) + (1 - n_i)\log(1 - p_i(\vec{y})) \quad (1)$$

where the first term contributes only when the given pixel in the sampled frame takes the value of 1, while the second term contributes when the pixel is 0 and weighs the probability of the pixel to be off. This likelihood definition allows for taking into account both activated and non-activated pixels. The prefactor $1/N_{\text{pixels}}$ normalises the $BCE$ to an average contribution per pixel, keeping its scale independent of the number of pixels entering the comparison and without affecting the position of the minimum with respect to $\vec{y}$. For each one, the $BCE$ was computed with respect to the PDF at every scanned position $\vec{y}$, and the position yielding the lowest $BCE$ value was selected as the best-fit estimate. We refer to this approach as the "likelihood" method.

Since the testable resolution of the likelihood method depends on the distance between two adjacent scanned positions, the calibration sample was augmented within a smaller portion of the volume, which was sampled with a higher density of points. At the depths of 245 mm and 345 mm from the sensor, 101 16-bit images were taken respectively, with a spacing of 0.1 mm. These were used to determine the lateral resolution. For the depth

resolution, data points with a spacing of 1 mm along the optical axis, between a 230 mm and 450 mm distance from the sensor, were gathered. Each of these intensity images was used as an empirical PDF to infer the position of the point light source, and the corresponding sampled low-photon-count datasets were generated as described above.

A depth resolution of 2.5 mm was achieved along with a lateral resolution of 0.1 mm. With the sampled photon-starved images, the depth and lateral residual decreased to, respectively, below 10 mm and 0.3 mm for five photons. Simulation studies have shown that the existence of degeneracies results in an oscillatory pattern in spatial resolution along both the lateral and depth directions when fewer than 30 counts are detected. This effect is due to statistical fluctuations and is not always reproducible in data, as too many steps of the movement stage and measurements would be needed. Below 10 counts, degeneracies arise also from the fact that points along a single line starting from the principal point of the main lens correspond to concentric images.

Before explicitly studying the capability of a PLATON-10cm module to detect GeV neutrinos, a simulation study was performed to evaluate the spatial resolution to a point light source corresponding to 1 MeV energy deposition. The post-processing method used to evaluate the spatial resolution for a point light source is the same as described in the "Image post-processing method" subsection of Methods of the main text. For a single-point scintillation light source, creating 10,000 photons, the 3D spatial resolution is 0.3 mm. If the number of cameras is reduced to two, the 3D spatial resolution becomes 0.8 mm.

For consistency, we compared the performance of the plenoptic cameras with classical cameras, obtained by removing the MLA and adapting the lens-to-sensor distance. The post-processing adopted for the classical cameras is the same as that used for plenoptic cameras, with the difference that now the pinhole is the main lens. While a single classical camera cannot precisely reconstruct the 3D position of the light source, the resolution of two orthogonal cameras is 2.4 mm, which is three times worse than that of the corresponding plenoptic system. Moreover, we note that two classical cameras fail to reconstruct the position of the light source for approximately 23% of the events, whereas all events are successfully reconstructed for two plenoptic cameras. The resolution for eight cameras, split into two orthogonal views, is approximately four times worse than that of the PLATON-10cm detector module, which utilises eight plenoptic cameras.

Moreover, in the eight-camera setup, 10% of the events with the worst resolution show an average residual of 3.3 mm, which is 4.2 times larger than the case of plenoptic cameras (0.8 mm). This means that plenoptic cameras exhibit a lower probability of making relatively large mistakes in estimating the light source position. The results of the simulation are shown in Supplementary Fig. 2.

Although an array of classical cameras is capable of reconstructing 3D point-like particle interactions in scintillator, the best performance is provided by an array of plenoptic cameras. Thus, we opted for this latter configuration as baseline for the PLATON detector module in this work.

## 3. Neural-network based reconstruction

Compared to convolutional neural networks (CNNs) [14, 15] or graph neural nets (GNNs) [16–18], which are constrained by local or explicitly defined receptive fields, transformers are not limited to such neighbourhoods and can integrate global spatial context more effectively. Recurrent neural networks (RNNs) [19–21] are also ill-suited for this task, as they assume a consistent sequential ordering, which does not naturally apply to unordered 2D photon hits [22]. In contrast, the transformer's attention mechanism provides the flexibility to learn arbitrary spatial dependencies, which is essential for resolving the complex and often ambiguous photon distributions resulting from neutrino interactions [23, 24]. An alternative class of models worth considering is Neural Radiance Fields (NeRFs) [25], which learn continuous volumetric scene representations from dense multi-view RGB data using ray-based modelling. While NeRFs have proven effective for reconstructing 3D geometry from 2D projections, their direct application to our setting is limited. SPAD sensors produce extremely sparse, binary detections, lacking the dense photometric supervision and calibrated viewing geometries that NeRFs rely on. Furthermore, NeRFs are primarily designed for photorealistic view synthesis, whereas

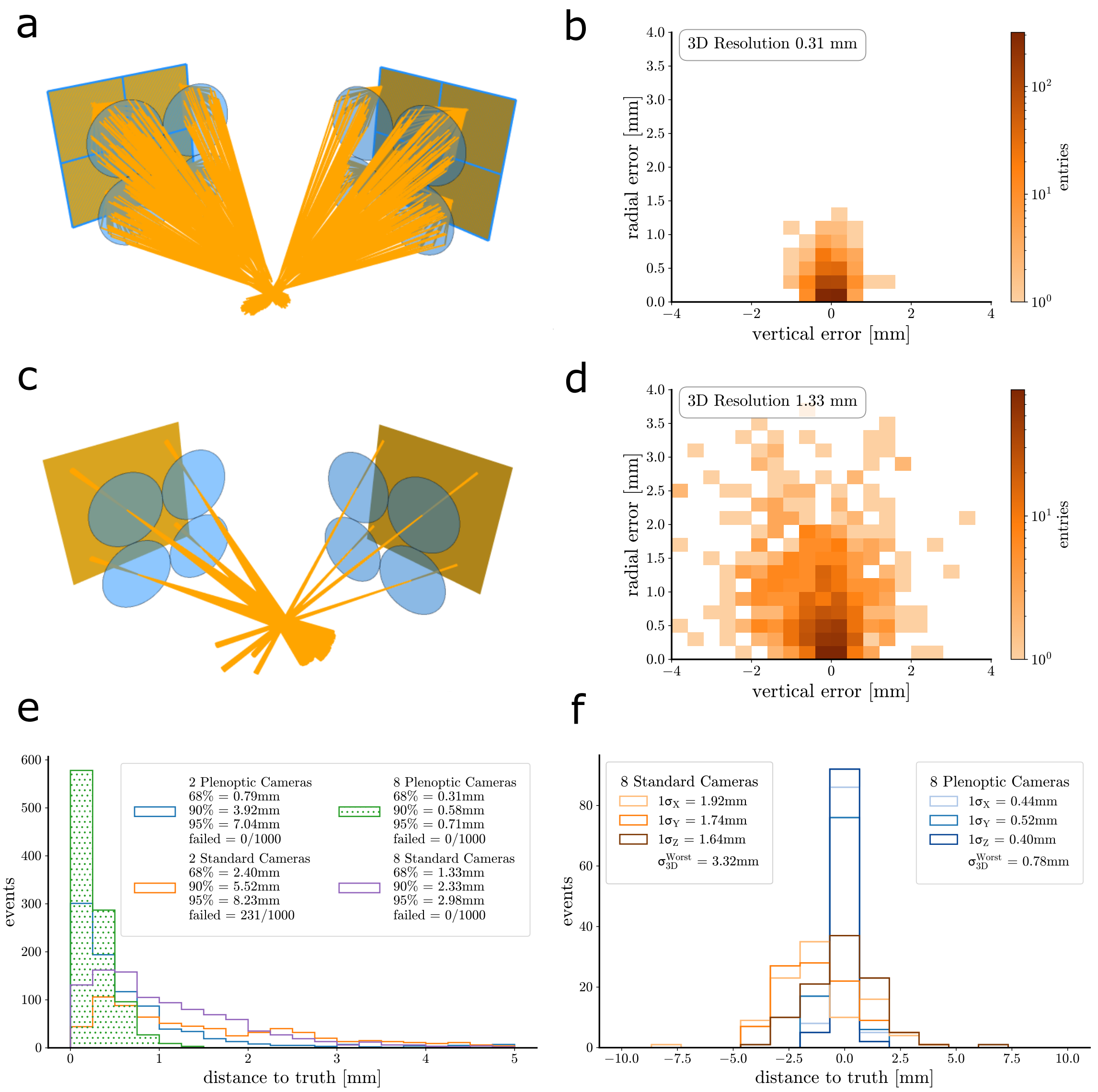


**Supplementary Fig. 2**: **Comparison of the performance between plenoptic and standard camera versions of the PLATON detector concept.** Event reconstruction of a simulated point-like light source with eight plenoptic **a** and classical **c** cameras. The spatial residual of the reconstructed vertical position and the cylindrical radial distance from the true origin of photons for eight plenoptic in **b** and standard in **d** cameras. **e** The distributions of 3D spatial residual, i.e., the Euclidean distance between the truth and reconstructed position of the source for 1,000 simulated events for eight and two orthogonal cameras for plenoptic and classical cameras. The legend displays the values of the 68%, 90% and 95% percentiles of the one-sided distribution as well as the number of events for which the post-processing failed. **f** The distribution of the residual of the worst reconstructed 10% of events is shown for both plenoptic (cold-coloured lines) and classical (warm-coloured lines) cameras. The peeling method, described in the “Image post-processing method” subsection of Methods of the main text, is not applied to either standard or classical cameras.

our goal is to recover the underlying spatial topology of particle interactions from non-image-based, unordered data. As such, although NeRF-inspired techniques may hold promise for future work [26, 27], they would require substantial re-engineering to address the unique challenges of our domain. Moreover, more traditional reconstruction methods are discarded due to the multi-dimensional complexity of the problem and the large number of sparse photons involved. These methods often lack the flexibility and computational efficiency required to process and interpret the intricate patterns of photon detection necessary for accurate 3D reconstruction.

The quadratic complexity $O(n^2)$ of the standard transformer's attention mechanism poses computational challenges for processing events with a large number of photons. To overcome this, we use a custom-adapted version of BigBird's sparse attention [28], which combines global, window, and random attention within blocks of tokens to achieve linear complexity $O(n)$ while effectively modelling long-range dependencies. Global attention allows specific tokens to attend to all others, window attention limits tokens to attend only to their neighbours, and random attention randomly connects tokens. This hybrid approach allows the model to handle long sequences while being computationally efficient. In particular, we based our code on HuggingFace's implementation [29, 30]. Specifically, the BigBird configuration used in our model includes a block size of 64 tokens, with custom modifications to the attention mechanism. Unlike the original BigBird implementation, our configuration does not utilise window attention; each token attends only to itself and not its neighbours (in the sequence space) or tokens within the same block unless that block is selected through random attention. Additionally, global attention is restricted to the global tokens within the first block and does not extend to all tokens in the block. The random attention spans 4 blocks, a value chosen specifically for this setup. These decisions in the attention mechanism arise from the fact that we are not working with true sequential data; the ordering of photons in the input sequence is arbitrary, and we aim to avoid the model learning spurious correlations or patterns from this ordering. Our transformer encoder architecture comprises 12 attention layers, a hidden size of 384 units, GELU activations [31], and 12 attention heads. Shuffling the photon sequence mitigates the risk of the network learning undesirable block patterns.

The Mean Squared Error (MSE) was our first choice as a loss function. It minimises the squared difference between the predicted and true 3D origins of photons. Formally, it is defined as

$$\mathcal{L}_{\text{MSE}} = \frac{1}{N} \sum_{i=1}^{N} \left\| \mathbf{y}_i - \hat{\mathbf{y}}_i \right\|^2 , \tag{2}$$

where $\mathbf{y}_i$ and $\hat{\mathbf{y}}_i$ denote the true and predicted 3D coordinates of the $i$-th photon origin, and $N$ is the total number of photons.

Initial experiments, however, revealed convergence difficulties due to inherent ambiguities in photon patterns. To mitigate this, we incorporated the Chamfer Distance (CD), commonly used in the point cloud reconstruction literature. CD relaxes the strict point-to-point correspondence by minimising the distance between each reconstructed point and its closest true point (and vice versa) and is given by:

$$\mathcal{L}_{\text{CD}} = \frac{1}{|\hat{\mathbf{Y}}|} \sum_{\hat{\mathbf{y}} \in \hat{\mathbf{Y}}} \min_{\mathbf{y} \in \mathbf{Y}} \left\| \hat{\mathbf{y}} - \mathbf{y} \right\|^2 + \frac{1}{|\mathbf{Y}|} \sum_{\mathbf{y} \in \mathbf{Y}} \min_{\hat{\mathbf{y}} \in \hat{\mathbf{Y}}} \left\| \mathbf{y} - \hat{\mathbf{y}} \right\|^2 . \tag{3}$$

where $\mathbf{Y}$ and $\hat{\mathbf{Y}}$ are the sets of true and predicted 3D points, respectively.

We use CD instead of the Earth Mover's Distance (EMD) [32] due to EMD's higher computational complexity and limitations in preserving the fidelity of detailed structures [33]. This approach facilitates learning the overall shape of the neutrino interaction rather than precisely backtracking each individual photon ray. A known limitation of CD is that it does not account for local variations in point density. In regions with low ground-truth density, a single true point may serve as the nearest neighbour for many predicted points, leading to an over-representation of these sparse areas in the loss, while regions with denser ground truth are under-represented. To overcome this limitation, the Density-Aware Chamfer Distance (DCD) [33] is adopted. The DCD augments the CD loss by incorporating a density-aware weighting scheme along with exponential mapping.

For each predicted point $\hat{y} \in \hat{\mathbf{Y}}$, denote its nearest neighbour in the true set $\mathbf{Y}$ and the corresponding squared distance by

$$y^*(\hat{y}) = \arg\min_{y \in \mathbf{Y}} \|\hat{y} - y\|^2, \quad d(\hat{y}) = \|\hat{y} - y^*(\hat{y})\|^2.$$

For each true point $y \in \mathbf{Y}$, denote its nearest neighbour in the predicted set $\hat{\mathbf{Y}}$ and the corresponding squared distance by

$$\hat{y}^*(y) = \arg\min_{\hat{y} \in \hat{\mathbf{Y}}} \|y - \hat{y}\|^2, \quad d(y) = \|y - \hat{y}^*(y)\|^2.$$

In the implementation, the number of times a point is queried is used for density weighting. Let $n\big(y^*(\hat{y})\big)$ be the number of predicted points that select $y^*(\hat{y})$ as their nearest neighbour, and $n\big(\hat{y}^*(y)\big)$ be the number of true points for which $\hat{y}^*(y)$ is the nearest neighbour. With a small constant $\varepsilon$ (i.e., $1 \times 10^{-12}$) and an exponent $n_\lambda$ (set to 0.5 in our experiments), the weights are given by

$$w(\hat{y}) = \frac{\gamma_{y\hat{y}}}{\big(n\big(y^*(\hat{y})\big)^{n_\lambda} + \varepsilon\big)}, \quad w(y) = \frac{\gamma_{\hat{y}y}}{\big(n\big(\hat{y}^*(y)\big)^{n_\lambda} + \varepsilon\big)}.$$

Here, the regularisation factors balance the contributions from the two sets and are defined as

$$\gamma_{y\hat{y}} = \frac{n_y}{n_{\hat{y}}}, \quad \gamma_{\hat{y}y} = \frac{n_{\hat{y}}}{n_y}$$

with $n_{\hat{y}} = |\hat{\mathbf{Y}}|$ and $n_y = |\mathbf{Y}|$.

An exponential mapping with scaling factor $\alpha$ (set to 40 in our experiments) is applied to the squared distances, so that

$$e^{-\alpha\, d(\hat{y})} \quad \text{and} \quad e^{-\alpha\, d(y)}$$

map each squared distance to the interval $(0, 1)$. Consequently, the Density-Aware Chamfer Distance loss is expressed as

$$\mathcal{L}_{\text{DCD}} = \frac{1}{2}\Bigg(\frac{1}{|\hat{\mathbf{Y}}|}\sum_{\hat{y} \in \hat{\mathbf{Y}}}\Big(1 - w(\hat{y})\, e^{-\alpha\, d(\hat{y})}\Big) + \frac{1}{|\mathbf{Y}|}\sum_{y \in \mathbf{Y}}\Big(1 - w(y)\, e^{-\alpha\, d(y)}\Big)\Bigg). \tag{4}$$

For small distances, the contribution is approximately linear; for larger distances, the contribution saturates, thereby reducing the influence of outliers. The total loss applied to the transformer encoder layers comprises both the DCD and a weighted MSE loss, ensuring a balanced learning process. Deep supervision [34, 35] is applied by computing the loss at each encoder layer with exponentially increasing weights. For a transformer with $l$ encoder layers, the total loss is

$$\mathcal{L}_{\text{total}} = \sum_{i=1}^{l} \frac{1}{2^{(l-i)}} \left(\mathcal{L}_{\text{DCD}} + \beta\, \mathcal{L}_{\text{MSE}}\right), \tag{5}$$

where $\beta$ is a scalar weight that balances the MSE loss with the DCD loss (set to 1 in our experiments), and the factor $\frac{1}{2^{(l-i)}}$ assigns greater weight to losses computed at deeper layers.

This combined loss function enables learning both the overall geometry and the fine-grained spatial photon distribution effectively, leading to improved convergence and reconstruction quality.

The model was implemented in Python 3.12.2 using PyTorch 2.5.1 [36] and PyTorch Lightning 2.4.0 [37], and trained on a single NVIDIA H100 GPU with 94 GB of memory. The training was conducted over 200 epochs with a batch size or 64 and a maximum input sequence length capped at 1024, as previously specified. The optimiser employed was AdamW [38, 39], with a learning rate initialised at $2 \times 10^{-4}$, $\beta_1 = 0.9$, $\beta_2 = 0.95$, and a weight decay of $10^{-4}$. The learning rate schedule consisted of a single warm-up epoch, followed by cosine annealing over the remaining 199 epochs, decaying smoothly to zero. Hyperparameter optimisation was conducted using the Optuna framework, enabling efficient search space exploration through automated sampling and pruning strategies [40]. No dropout was applied, as empirical evidence indicated a detrimental effect on the regression of precise continuous values. The generalisation capacity typically provided by dropout was instead achieved through the stochastic sampling of photons per event. Given the large number of possible photon combinations, the likelihood of the model encountering the same subset of inputs across epochs is negligible. All results presented in the "Simulated particle detection with the PLATON-10cm detector" subsection of Methods of the main text were obtained on the held-out test set from a 60%/10%/30% train/validation/test split, respectively.

## 4. Details of the neutrino simulation analysis

### Post-processing

The neutrino event post-processing consists of the following steps: first, a significant fraction of photosensor dark counts are rejected by signal coincidence in a time window of 10 ns; the 2D images collected by the eight cameras and the muon smeared exiting position (mean = 0 mm, std = 1 mm) and direction (mean = 0 radians, std = 0.05 radians) are given as input to the neural network (NN). No timing information is passed to the NN, which instead returns the 3D origin of each photon associated with a count in the scintillator volume. The result is a cloud of points ("photon origins") that indicate the origin of the scintillation photons along the trajectory of every charged particle. This is illustrated in Fig. 4 of the main text.

### Pattern recognition

The reconstruction of PLATON 3D images differs from what data processing in particle physics experiments typically requires, and is illustrated in Fig. 7b of the main text. The clustering algorithm is based on Gaussian Mixture Model (GMM) [41]: a probabilistic model that represents complex data as a combination of simpler sub-distributions, Gaussian in this case, of underlying hidden classes. The optimal number of clusters was selected using the elbow method, a heuristic that identifies the point beyond which increasing the number of clusters yields only marginal improvements in model performance. In a second step, principal component analysis (PCA) was used to merge clusters into tracks, each assigned to a different particle. This process iterated through all pairs of clusters, merging two clusters if they were both collinear and sufficiently close to each other. Once all the particle tracks of the event were obtained, the vertex of the neutrino interaction was reconstructed. Since the muon is assumed to be determined by an external detector, we use its exiting point on the scintillator surface to assign one of the tracks to the muon. Finally, the extreme of the muon track opposite to its exiting point is identified as the neutrino interaction vertex. In some cases, multiple scattering can break the muon track into different consecutive segments. To overcome this issue, the clustering algorithm is rerun with slightly relaxed conditions, potentially adding a new cluster to the muon track, until a connected but non-collinear cluster is found. If the muon does not escape the scintillator volume, the longest reconstructed track is assigned to it, and the end with the largest number of connected clusters is identified as the interaction vertex. If the two track ends exhibit an equal number of connected clusters, the point forming the smaller angle with the connected cluster is selected as the interaction vertex.

### Selection of neutrino events

The CC $1\mu 0\pi 1p$ sample is the most common final-state topology below 1 GeV, observed in experiments such as T2K or Hyper-Kamiokande, since it is dominated by CC quasi-elastic (CCQE) interactions. Another type of final-state topology is named 2 protons - 2 holes (2p2h) and can be induced by three main types of processes: meson exchange current, where the momentum transfer is shared between two nucleons via the exchange of a virtual meson [42]; short-range nucleon-nucleon correlations (SRC) [43], where the neutrino interacts with a nucleon that is part of a correlated nucleon-nucleon pair and both nucleons are knocked out of the nucleus; FSI, with a nucleon that knocks out a second nucleon. 2p2h events are less frequent ($\sim 8\%$) than CCQE interactions ($\sim 43\%$), but are notoriously affected by significant systematic uncertainties in the modelling of the process [44–46]. Thus, the correct modelling of 2p2h interactions is of vital importance for the ongoing precision measurements of neutrino oscillations [47–50] and, in particular, for the next-generation LBL experiments that will search for leptonic charge-parity violation [51–53].

The accurate identification of the type of each particle produced by the neutrino interaction relies on the precise measurement of the energy loss along its trajectory. For instance, the detection of the Bragg peak of particles stopping in the scintillator ensures unambiguous particle identification (PID). This feature is enhanced by the fact that most of the muons are produced with a momentum peaked around 0.5 GeV/c with a long tail above 1 GeV, while protons are expected dominantly below 1 GeV/c with a peak around 300 MeV/c. The reconstructed distribution of the

energy loss along the track of protons (often stopping in the scintillator) and muons is shown in Fig. 5 of the main text. To identify the particle type associated with each reconstructed track independently, a boosted decision tree (BDT) classifier was employed using the XGBoost algorithm [54]. The kinematic variables used as input features to the BDT include: the variance of the number of photons per mm, the total number of photons in the track, the maximum number of photons per mm, the minimum number of photons per mm, and the mean number of photons per mm.

### Selection of neutrino events with a system of classical cameras

The same study has been conducted with a configuration of the PLATON-10cm module where a conventional optical system is obtained by removing the MLA. It was found that, although a system based on standard cameras exhibits inferior spatial resolution compared to an equivalent system employing plenoptic cameras (see above), even orthogonal arrays of conventional cameras instrumented with SPAD array sensors can achieve very good performance within a PLATON-10cm module over a range of 10 cm. On the other hand, the drop in selection efficiency below 50% occurs at 215 MeV/$c$ (corresponding to a 3.5 mm proton range) with the MLA, and at 230 MeV/$c$ (corresponding to a 5 mm proton range) without the MLA. A similar difference can be found in the neutrino vertex resolution and the proton angular resolution: the vertex resolution drops from 0.4 mm to 0.6 mm, while the angular resolution falls from 1.5° to 1.8°. This is in line with the results shown above. Without the MLA, the efficiency and the purity are, respectively, 6% and 5% lower compared to the plenoptic configuration. It is worth noting that the PLATON-10cm module is required to cover a depth of field of 10 cm; therefore, it is plausible that eight classical cameras combined can achieve a reasonably good depth resolution.

## References


[1] Allison, J., Amako, K., Apostolakis, J., Araujo, H., Arce Dubois, P., Asai, M., Barrand, G., Capra, R., Chauvie, S., Chytracek, R., *et al.*: Geant4 developments and applications. IEEE Transactions on Nuclear Science **53**, 270–278 (2006) https://doi.org/10.1109/tns.2006.869826

[2] Agostinelli, S., Allison, J., Amako, K., Apostolakis, J., Araujo, H., Arce, P., Asai, M., Axen, D., Banerjee, S., Barrand, G., *et al.*: Geant4—a simulation toolkit. Nuclear Instruments and Methods in Physics Research Section A: Accelerators, Spectrometers, Detectors and Associated Equipment **506**, 250–303 (2003) https://doi.org/10.1016/s0168-9002(03)01368-8

[3] Allison, J., Amako, K., Apostolakis, J., Arce, P., Asai, M., Aso, T., Bagli, E., Bagulya, A., Banerjee, S., Barrand, G., *et al.*: Recent developments in geant4. Nuclear Instruments and Methods in Physics Research Section A: Accelerators, Spectrometers, Detectors and Associated Equipment **835**, 186–225 (2016) https://doi.org/10.1016/j.nima.2016.06.125

[4] Eljen: Green Emitting Plastic Scintillator EJ-260 and EJ-262. (2016). https://eljentechnology.com/products/plastic-scintillators/ej-260-ej-262

[5] Abusleme, A., *et al.*: JUNO physics and detector. Prog. Part. Nucl. Phys. **123**, 103927 (2022) https://doi.org/10.1016/j.ppnp.2021.103927 arXiv:2104.02565 [hep-ex]

[6] An, F.P., *et al.*: Observation of electron-antineutrino disappearance at Daya Bay. Phys. Rev. Lett. **108**, 171803 (2012) https://doi.org/10.1103/PhysRevLett.108.171803 arXiv:arXiv:1203.1669 [hep-ex]

[7] Abe, K., et al.: Measurements of neutrino oscillation in appearance and disappearance channels by the T2K experiment with 6.6x10ˆ20 protons on target. Physical Review D **91** (2015) https://doi.org/10.1103/physrevd.91.072010

[8] T2K Beam Group, W.: Neutrino Beam Flux Prediction 2016. The T2K Experiment (2016). https://t2k-experiment.org/results/neutrino-beam-flux-prediction-2016/

[9] Abe, K., *et al.*: T2K neutrino flux prediction. Phys. Rev. D **87**(1), 012001 (2013) https://doi.org/10.1103/PhysRevD.87.012001 arXiv:1211.0469 [hep-ex]. [Addendum: Phys.Rev.D 87, 019902 (2013)]

[10] Hayato, Y., Pickering, L.: The neut neutrino interaction simulation program library. The European Physical Journal Special Topics **230**, 4469–4481 (2021) https://doi.org/10.1140/epjs/s11734-021-00287-7

[11] Tanabashi, M., Hagiwara, K., Hikasa, K., Nakamura, K., Sumino, Y., Takahashi, F., Tanaka, J., Agashe, K., Aielli, G., Amsler, C., *et al.*: Review of particle physics. Phys. Rev. D **98**, 030001 (2018) https://doi.org/10.1103/PhysRevD.98.030001

[12] Kolanoski, H., Wermes, N.: Particle Detectors. Oxford University Press, Oxford, UK (2020)

[13] Ulku, A.C., Bruschini, C., Antolovic, I.M., Kuo, Y., Ankri, R., Weiss, S., Michalet, X., Charbon, E.: A 512 ×512 spad image sensor with integrated gating for widefield flim. IEEE Journal of Selected Topics in Quantum Electronics **25**, 1–12 (2019) https://doi.org/10.1109/jstqe.2018.2867439

[14] LeCun, Y., Boser, B., Denker, J.S., Henderson, D., Howard, R.E., Hubbard, W., Jackel, L.D.: Backpropagation applied to handwritten zip code recognition. Neural Computation **1**(4), 541–551 (1989) https://doi.org/10.1162/neco.1989.1.4.541

[15] Li, Z., Liu, F., Yang, W., Peng, S., Zhou, J.: A survey of convolutional neural networks: Analysis, applications, and prospects. IEEE Transactions on Neural Networks and Learning Systems **33**(12), 6999–7019 (2022) https://doi.org/10.1109/TNNLS.2021.3084827

[16] Scarselli, F., Gori, M., Tsoi, A.C., Hagenbuchner, M., Monfardini, G.: The graph neural network model. IEEE Transactions on Neural Networks **20**(1), 61–80 (2009) https://doi.org/10.1109/TNN.2008.2005605

[17] Wu, Z., Pan, S., Chen, F., Long, G., Zhang, C., Yu, P.S.: A comprehensive survey on graph neural networks. IEEE Transactions on Neural Networks and Learning Systems **32**(1), 4–24 (2021) https://doi.org/10.1109/TNNLS.2020.2978386

[18] Zhou, J., Cui, G., Hu, S., Zhang, Z., Yang, C., Liu, Z., Wang, L., Li, C., Sun, M.: Graph neural networks: A review of methods and applications. AI Open **1**, 57–81 (2020) https://doi.org/10.1016/j.aiopen.2021.01.001

[19] Jordan, M.I.: Chapter 25 - serial order: A parallel distributed processing approach. In: Donahoe, J.W., Packard Dorsel, V. (eds.) Neural-Network Models of Cognition. Advances in Psychology, vol. 121, pp. 471–495. North-Holland, Amsterdam, Netherlands (1997). https://doi.org/10.1016/S0166-4115(97)80111-2 . https://www.sciencedirect.com/science/article/pii/S0166411597801112

[20] Jain, L.C., Medsker, L.R.: Recurrent Neural Networks: Design and Applications, 1st edn. CRC Press, Inc., USA (1999)

[21] Sherstinsky, A.: Fundamentals of recurrent neural network (RNN) and long short-term memory (LSTM) network. Physica D: Nonlinear Phenomena **404**, 132306 (2020) https://doi.org/10.1016/j.physd.2019.132306

[22] Xiao, C., Sun, J.: Recurrent Neural Networks (RNN), pp. 111–135. Springer, Cham (2021). https://doi.org/10.1007/978-3-030-82184-5_7 . https://doi.org/10.1007/978-3-030-82184-5_7

[23] Islam, S., Elmekki, H., Elsebai, A., Bentahar, J., Drawel, N., Rjoub, G., Pedrycz, W.: A comprehensive survey on applications of transformers for deep learning tasks. Expert Systems with Applications **241**, 122666 (2024) https://doi.org/10.1016/j.eswa.2023.122666

[24] Khan, S., Naseer, M., Hayat, M., Zamir, S.W., Khan, F.S., Shah, M.: Transformers in vision: A survey. ACM Comput. Surv. **54**(10s) (2022) https://doi.org/10.1145/3505244

[25] Mildenhall, B., Srinivasan, P.P., Tancik, M., Barron, J.T., Ramamoorthi, R., Ng, R.: NeRF: Representing Scenes as Neural Radiance Fields for View Synthesis (2020). https://arxiv.org/abs/2003.08934

[26] Yu, A., Ye, V., Tancik, M., Kanazawa, A.: pixelNeRF: Neural Radiance Fields from One or Few Images (2021). https://arxiv.org/abs/2012.02190

[27] Niemeyer, M., Barron, J.T., Mildenhall, B., Sajjadi, M.S.M., Geiger, A., Radwan, N.: RegNeRF: Regularizing Neural Radiance Fields for View Synthesis from Sparse Inputs (2021). https://arxiv.org/abs/2112.00724

[28] Zaheer, M., Guruganesh, G., Dubey, A., Ainslie, J., Alberti, C., Ontanon, S., Pham, P., Ravula, A., Wang, Q., Yang, L., Ahmed, A.: Big Bird: Transformers for Longer Sequences (2021). https://arxiv.org/abs/2007.14062

[29] Wolf, T., Debut, L., Sanh, V., Chaumond, J., Delangue, C., Moi, A., Cistac, P., Rault, T., Louf, R., Funtowicz, M., et al.: HuggingFace's Transformers: State-of-the-art Natural Language Processing (2020). https://arxiv.org/abs/1910.03771

[30] HuggingFace: BigBird model (2024). https://github.com/huggingface/transformers/tree/v4.42.0/src/transformers/models/big_bird

[31] Hendrycks, D., Gimpel, K.: Gaussian Error Linear Units (GELUs) (2023). https://arxiv.org/abs/1606.08415

[32] Rubner, Y., Tomasi, C., Guibas, L.J.: A metric for distributions with applications to image databases. In: Sixth International Conference on Computer Vision (IEEE Cat. No.98CH36271), pp. 59–66 (1998). https://doi.org/10.1109/ICCV.1998.710701

[33] Wu, T., Pan, L., Zhang, J., Wang, T., Liu, Z., Lin, D.: Density-aware Chamfer Distance as a Comprehensive Metric for Point Cloud Completion (2021). https://arxiv.org/abs/2111.12702

[34] Lee, C.-Y., Xie, S., Gallagher, P., Zhang, Z., Tu, Z.: Deeply-Supervised Nets (2014). https://arxiv.org/abs/1409.5185

[35] Li, R., Wang, X., Huang, G., Yang, W., Zhang, K., Gu, X., Tran, S.N., Garg, S., Alty, J., Bai, Q.: A Comprehensive Review on Deep Supervision: Theories and Applications (2022). https://arxiv.org/abs/2207.02376

[36] Paszke, A., Gross, S., Massa, F., Lerer, A., Bradbury, J., Chanan, G., Killeen, T., Lin, Z., Gimelshein, N., Antiga, L., *et al.*: PyTorch: An imperative style, high-performance deep learning library. In: Advances in Neural Information Processing Systems 32, pp. 8024–8035. Curran Associates, Inc., Red Hook, NY, USA (2019). http://papers.neurips.cc/paper/9015-pytorch-an-imperative-style-high-performance-deep-learning-library.pdf

[37] Falcon, W., The PyTorch Lightning team: PyTorch Lightning (2019). https://doi.org/10.5281/zenodo.3828935 . https://github.com/Lightning-AI/lightning

[38] Kingma, D.P., Ba, J.: Adam: A Method for Stochastic Optimization (2017). https://arxiv.org/abs/1412.6980

[39] Loshchilov, I., Hutter, F.: Decoupled Weight Decay Regularization (2019). https://arxiv.org/abs/1711.05101

[40] Akiba, T., Sano, S., Yanase, T., Ohta, T., Koyama, M.: Optuna: A next-generation hyperparameter optimization framework. In: Proceedings of the 25th ACM SIGKDD International Conference on Knowledge Discovery and Data Mining (2019)

[41] Dempster, A.P., Laird, N.M., Rubin, D.B.: Maximum likelihood from incomplete data via the em algorithm. Journal of the Royal Statistical Society: Series B (Methodological) **39**(1), 1–22 (2018) https://doi.org/10.1111/j.2517-6161.1977.tb01600.x https://academic.oup.com/jrsssb/article-pdf/39/1/1/49117094/jrsssb_39_1_1.pdf

[42] Alvarez-Ruso, L., Athar, M.S., Barbaro,

M.B., Cherdack, D., Christy, M.E., Coloma, P., Donnelly, T.W., Dytman, S., Gouvêa, A., Hill, R.J., *et al.*: Nustec1 1neutrino scattering theory experiment collaboration http://nustec.fnal.gov. white paper: Status and challenges of neutrino–nucleus scattering. Progress in Particle and Nuclear Physics **100**, 1–68 (2018) https://doi.org/10.1016/j.ppnp.2018.01.006

[43] Hen, O., Miller, G.A., Piasetzky, E., Weinstein, L.B.: Nucleon-nucleon correlations, short-lived excitations, and the quarks within. Rev. Mod. Phys. **89**, 045002 (2017) https://doi.org/10.1103/RevModPhys.89.045002

[44] Gran, R., Nieves, J., Sanchez, F., Vicente Vacas, M.J.: Neutrino-nucleus quasi-elastic and 2p2h interactions up to 10 GeV. Phys. Rev. D **88**(11), 113007 (2013) https://doi.org/10.1103/PhysRevD.88.113007 arXiv:arXiv:1307.8105 [hep-ph]

[45] Nieves, J., Sanchez, F., Ruiz Simo, I., Vicente Vacas, M.J.: Neutrino Energy Reconstruction and the Shape of the CCQE-like Total Cross Section. Phys. Rev. D **85**, 113008 (2012) https://doi.org/10.1103/PhysRevD.85.113008 arXiv:arXiv:1204.5404 [hep-ph]

[46] Sobczyk, J.E., Nieves, J., Sánchez, F.: Exclusive final state hadron observables from neutrino-nucleus multi-nucleon knockout. Phys. Rev. C **102**(2), 024601 (2020) https://doi.org/10.1103/PhysRevC.102.024601 arXiv:arXiv:2002.08302 [nucl-th]

[47] Acero, M.A., *et al.*: Adjusting neutrino interaction models and evaluating uncertainties using nova near detector data. The European Physical Journal C **80**(12), 1119 (2020) https://doi.org/10.1140/epjc/s10052-020-08577-5

[48] Abratenko, P., *et al.*: New CC0$\pi$ GENIE model tune for MicroBooNE. Phys. Rev. D **105**, 072001 (2022) https://doi.org/10.1103/PhysRevD.105.072001

[49] Abe, K., *et al.*: Measurement of neutrino and antineutrino oscillations by the T2K experiment including a new additional sample of $\nu_e$ interactions at the far detector. Phys. Rev. D **96**, 092006 (2017) https://doi.org/10.1103/PhysRevD.96.092006

[50] Abe, K., *et al.*: Measurements of neutrino oscillation parameters from the T2K experiment using $3.6 \times 10^{21}$ protons on target. The European Physical Journal C **83**(9), 782 (2023) https://doi.org/10.1140/epjc/s10052-023-11819-x

[51] Proto-Collaboration, H.-K., :, Abe, K., Abe, K., Aihara, H., Aimi, A., Akutsu, R., Andreopoulos, C., Anghel, I., Anthony, L.H.V., Antonova, M., Ashida, Y., et al.: Hyper-Kamiokande Design Report (2018). https://arxiv.org/abs/1805.04163

[52] Abi, B., *et al.*: Long-baseline neutrino oscillation physics potential of the DUNE experiment. Eur. Phys. J. C **80**(10), 978 (2020) https://doi.org/10.1140/epjc/s10052-020-08456-z arXiv:arXiv:2006.16043 [hep-ex]

[53] Abud Abed, A., *et al.*: Low exposure long-baseline neutrino oscillation sensitivity of the DUNE experiment. Phys. Rev. D **105**(7), 072006 (2022) https://doi.org/10.1103/PhysRevD.105.072006 arXiv:2109.01304 [hep-ex]

[54] Chen, T., Guestrin, C.: Xgboost: A scalable tree boosting system. In: Proceedings of the 22nd ACM SIGKDD International Conference on Knowledge Discovery and Data Mining. KDD '16, pp. 785–794. Association for Computing Machinery, New York, NY, USA (2016). https://doi.org/10.1145/2939672.2939785 . https://doi.org/10.1145/2939672.2939785
